# A case study comparing anonymized and synthetic health insurance claims data for medication safety assessments

**DOI:** 10.1038/s41746-026-02622-5

**Published:** 2026-04-13

**Authors:** Mehmed Halilovic, Thierry Meurers, Marco Alibone, Marion Ludwig, Paul Tiwald, Nico Sieberg-Riedel, Steven Wolter, Lisa Kühnel, Steffen Hess, Fabian Prasser, Karen Otte

**Affiliations:** 1 Medical Informatics Group, Berlin Institute of Health at Charité – Universitätsmedizin Berlin, Berlin, Germany; 2 InGef - Institut für angewandte Gesundheitsforschung Berlin GmbH, Berlin, Germany; 3 MOSTLY AI, Vienna, Austria; 4 Forschungsdatenzentrum Gesundheit, Bundesinstitut für Arzneimittel und Medizinprodukte, Bonn, Germany

**Keywords:** Health care, Mathematics and computing, Medical research, Risk factors

## Abstract

Synthetic data generation is increasingly proposed as an alternative to classical anonymization for sharing health data. We compared concrete applications of both approaches on a small, high-dimensional health claims dataset, assessing their impact on fidelity, reproducibility of study outcomes, and privacy risks. To reflect different sharing contexts, we considered a context-independent, higher-risk scenario with no assumptions about potential attacks, and a context-dependent, lower-risk scenario informed by threat modeling. Analyses on anonymized and synthetic data yielded results similar to those from the original study data, but came at the cost of higher uncertainty when estimating hazard ratios. As expected, higher data utility and fidelity were related to higher privacy risks. Our findings provide a reusable workflow and comparative insights into anonymization and synthetization and show that both methods are valuable means to lower privacy risks in data sharing scenarios but verifying results on the original data should be done whenever possible.

## Introduction

Data play a significant role in advancing biomedical research and improving the quality of healthcare. Health claims data, in particular, can serve as a critical resource for evaluating healthcare utilization, real-world effectiveness of treatments, and patient outcomes^1^. By enabling studies of treatments in routine practice and across patient groups often underrepresented in clinical trials, these data help address evidence gaps, inform treatment recommendations and allow the identification of rare adverse events or side-effects of medication^2^. These data encompass detailed records of healthcare services provided, such as inpatient and outpatient care, pharmaceutical prescriptions, and medical procedures, along with associated costs. Sharing such data openly can provide a valuable resource for future research^3^.

However, health data is subject to strict data protection regulations to ensure the privacy of individuals^4^. The use of data protection mechanisms like anonymization or synthetization can reduce an individual’s likelihood of re-identification^5,6^. Rules and guidance on anonymization can be found in the US HIPAA Privacy Rule^7^ and the European General Data Protection Regulation (GDPR)^8^, as well as national implementations like the German Federal Data Protection Act^9^. Stronger privacy protection usually results in a decrease in the utility of the data, and both aspects must be balanced against each other^10^.

Major frameworks like GDPR and HIPAA leave the choice of the protection method to the data holder, given that certain risks to individuals represented in the data are protected against. For example, the Article 29 Working Party, the predecessor to today’s European Data Protection Board, identified three key risks that should be addressed: singling-out, the risk of isolating an individual within the dataset, linkability, the risk of connecting records in the dataset to other records concerning the same individual, and attribute inference, the risk of inferring additional information about an individual^11^.

Traditional data anonymization algorithms mitigate privacy risks by transforming data while trying to minimize the impact on its utility. These algorithms usually rely on formal privacy models, such as *k*-anonymity^12^ and *t*-closeness^13^, to define the level of protection needed in the anonymized data. To ensure these privacy protection levels, anonymization transforms the dataset, for example, through suppression, generalization, or microaggregation. A common approach involves applying uniform protection across all variables within a dataset, which may be overly conservative and can lead to substantial degradation of output data utility^10^. In contrast, regulatory guidelines^14–16^ provide recommendations on how to choose an appropriate set of variables to protect and offer guidance for privacy protection levels based on the data sharing context. This involves specific recommendations for variables that need to be protected, as well as recommendations for a case-by-case assessment. Additionally, the level of privacy protection needed is context-dependent, influenced by factors highlighted in such guidelines, such as the presumed motivation of potential adversaries and the intended sharing context (e.g., public release or restricted access).

Synthetic data generation presents a compelling alternative approach to anonymization for privacy-preserving data sharing^17^. It involves generating a completely new dataset, using mathematical models or algorithms trained on original data^18–20^. It aims to replicate the statistical properties of the original dataset without disclosing sensitive information on individuals, making it a promising approach with the potential to increase data accessibility. However, its practical application in the context of complex healthcare data and health claims data, which are characterized by intricate dependencies and correlations across variables, presents distinct challenges^21^. Preserving these relationships during the synthesis process is crucial to ensure that the resulting data retains its informative value and usability. Literature addressing synthetic data generation for secondary use of health claims data remains limited. Nevertheless, recent research has demonstrated that synthesizing health claims data is feasible, both in terms of maintaining utility and ensuring privacy, highlighting its potential for facilitating data sharing and enabling broader use of such data for research purposes^22^.

While anonymization benefits from established regulatory guidelines and recommendations, such standards don’t address synthetic data generation. At present, there is no universally accepted method to assess the level of privacy protection synthetic data provides^23^. Research indicates that synthetic data can still be vulnerable to disclosure, for example through membership inference attacks^24^, which try to infer whether a target record was part of the training set used for synthetization. To mitigate this problem, synthetic data generation methods may be tested empirically^25^, or include privacy guarantees, for example by incorporating Differential Privacy^26–28^. However, many studies investigating the use of synthetic data omit a detailed privacy assessment^23^. Even fewer studies directly compare anonymization and synthetization using real-world datasets and practical use cases^29,30^, and to our knowledge, none have specifically focused on health claims data.

In summary, there is limited empirical evidence on whether synthetic data outperforms classical anonymization techniques, particularly in the context of real-world, small and high-dimensional health datasets. Addressing this gap, and given the need for broader access to health claims data for research purposes, as highlighted in national and international discussions^31–33^, and recognizing that both anonymized and synthesized data can retain vulnerabilities or result in low-quality data if not carefully implemented and evaluated^24,34,35^, this study systematically addresses the practical application and comparative assessment of these two data protection methods. Rather than benchmarking anonymization and synthetization as entire method families, this study examines their concrete application on a challenging real-world health claims dataset. A previously published study on medication safety assessments offers an example of a realistic application of such data, as it performs a longitudinal analysis to investigate the risk of major bleeding and all-cause mortality of individuals with venous thromboembolisms (VTE) under oral anticoagulation and concomitant antiplatelet therapy^2^. In this work, we investigate the reproducibility of the findings from the original study when analyzing synthetic and anonymized versions of the dataset in the context of open data publication. Additionally, we assess the level of privacy protection these data versions offer. We approach this question by evaluating specific implementations of anonymization and synthetization across three core dimensions:

- Fidelity: How well are selected statistical properties of the original study dataset preserved?
- Utility: Can the results of a specific, previously published study investigating medication safety in venous thromboembolism (VTE) be reproduced?
- Privacy risk: How are privacy risks reduced in comparison to the original study dataset?

Importantly, we do this in a structured manner, investigating the concrete application of anonymization and synthetization, both within and outside the given use context. This results in a higher-risk scenario (i.e. context-independent), where no assumptions can be made about potential attacks, and a lower-risk scenario (i.e. context-dependent), where threat modeling is used to get a more realistic picture of privacy risks. It is well established that anonymization should be considered in the context of its use (see Five-Safes framework^36^). We examine each protection method under both the context-dependent and the context-independent scenario. This lets us estimate how a context-dependent anonymization holds up against an unrestricted adversary, and, conversely, reveals the downsides of overprotection by testing a generic (context-independent) anonymization in a context-dependent setting and how both compare to a (context-independent) synthetization. In each case, we assess privacy risk alongside utility to understand the trade-offs. The concepts of context-dependence and independence are also applicable to the utility evaluation of protected datasets. Here, the terms fidelity for a context-independent evaluation and utility for a context-dependent evaluation are established^37^.

To the best of our knowledge, this is the first comparative application of these privacy-preserving methods on a real-world health claims dataset using a systematic approach. Importantly, our framework includes a privacy assessment, utilizing the Anonymeter framework^25^ and shadow model-based membership inference attacks^24,38^, as well as realistic utility assessments based on a real-world study, addressing a critical shortcoming in synthetic data research, which often omits formal privacy evaluations^23^ and lacks real-world applications. In doing so, we outline a workflow for comparing applications of anonymization and synthetization along our proposed dimensions. This proposed workflow offers a structure that can be adapted to various datasets and reused in other contexts.

## Results

For this study, three protected datasets were created. These were derived from a health claims dataset which was created using one of the data sources utilized in the original VTE study^2^.

The study compares concomitant use of direct oral anticoagulants (DOACs) and antiplatelets with concomitant use of vitamin K antagonists (VKAs) and antiplatelets (see Methods section on Study data and dataset description for further details). The three protected datasets were context-independent anonymization, context-dependent anonymization, and context-independent synthetization. The context-independent anonymization dataset involved applying protection to all attributes. This is motivated by a higher-risk scenario in which the context of data use, organizational protection measures and data sharing settings cannot be reliably constrained, and thus no restriction is made on what an attacker could use as auxiliary information. The context-dependent anonymization dataset was created by applying protection to attributes identified through threat modeling. This is motivated by a lower-risk scenario, in which context of data use, organizational protection measures, and data sharing settings can be controlled. The context-independent synthetization dataset was created by applying synthetization to all attributes using the open-source framework by MOSTLY AI^39^.

See “Methods” section on Data sharing context, Data anonymization and Data synthetization for further details.

### Fidelity evaluation

To assess data fidelity, representing the structural similarity to the original study data, descriptive statistics across the original study, anonymized and synthesized datasets were compared.

Table 1 shows that the original study dataset, consisting of 39 attributes, contained 1727 records, with 1234 (71.5%) records in the DOAC and 493 (28.5%) in the VKA group. Context-independent anonymization suppressed 628 records completely, reducing the dataset to 1099 records and decreasing the number of DOAC cases to 881 (80.2%), causing a proportional shift between treatment groups of +8.7% towards DOAC. Context-dependent anonymization led to a smaller number of 394 suppressed records, leaving 1333 records, of which 968 (72.6%) were in the DOAC group. With synthetization, the generated dataset retained the total number of 1727 records, with 1260 (73.0%) in the DOAC group. For the demographic characteristic female, the proportion decreased from 43.4% in the original study dataset to 42.3% in the context-independent anonymization and 40.5% in the context-dependent anonymization, while in the synthetic dataset it increased to 46.8%. Mean age changed slightly from 74.7 years in the original study dataset to 75.15 years in context-independent anonymization, which is a relative increase of +0.60%. A smaller increase can be seen for context-dependent anonymization with +0.04% and for the synthetic dataset, a decrease of -0.86%. In summary, we see the largest data loss and proportional shifts in context-independent anonymization. The context-dependent anonymization shows data loss but retains proportions well, while the synthetization shows some proportional shifts.

**Table 1:** Study population and dataset characteristics

Parameter	Original study data	Independent anonymization	Dependent anonymization	Synthetization
Total records, *n (% of original)*	1727	1099 (63.6%)	1333 (77.2%)	1727 (100%)
**Treatment groups**				
- DOAC *n (% of group total)*	1234 (71.5%)	881 (80.2%)	968 (72.6%)	1260 (73.0%)
- VKA *n (% of group total)*	493 (28.5%)	218 (19.8%)	365 (27.4%)	467 (27.0%)
**Demographic characteristics**				
Female, *n (% of total records)*	750 (43.4%)	465 (42.3%)	540 (40.5%)	808 (46.8%)
Age, years, *mean (SD)*	74.70 (11.67)	75.15 (10.71)	74.73 (10.95)	74.06 (11.98)

The table summarizes the total number of records, treatment group composition, and basic demographic characteristics for the original, synthetic and anonymized datasets. DOAC indicates the direct oral anticoagulant treatment group, and VKA indicates the vitamin K antagonist treatment group. Age is reported as the mean with standard deviation (SD) for each dataset.

**Fig. 1:**
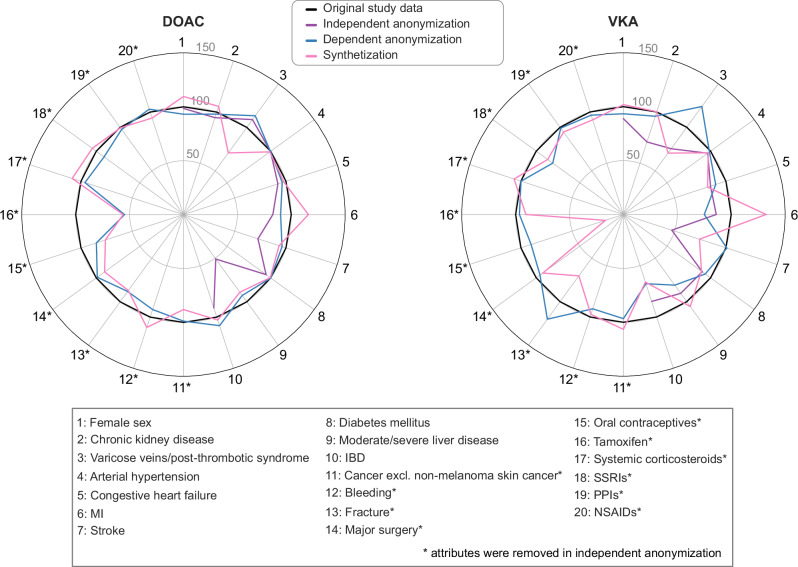
Comparison of prevalence of binary baseline patient covariates in the DOAC and VKA cohort. The polar chart compares the prevalence of binary baseline characteristics in the three protected datasets against the original study data. For each variable, the value from the original study data is normalized to a baseline of 100. The other datasets are plotted as a percentage of this original value, where closer alignment to the 100-mark indicates better preservation of the original prevalence. For clarity, the obesity flag was omitted as it had near-zero prevalence in the original study data, and all three protection methods removed it completely.

The polar charts in Fig. 1 visualize how well the anonymized and synthesized datasets retain data proportions of the 20 binary covariates. Since the anonymized datasets consist of fewer records, comparisons are based on relative distributions rather than absolute numbers of records. Hence, the total number of records was normalized to 100% within each dataset, and differences were expressed in percent. As expected, the context-independent anonymization starkly altered univariate distributions, and 10 of the shown attributes were completely removed. In contrast, the context-dependent anonymization and the synthetization largely maintain the original distributions of these attributes. However, larger differences between the original and these two protected datasets appear in 4 covariates (Varicose veins/post-thrombotic syndrome, myocardial infarction, oral contraception and tamoxifen). In the larger DOAC group, variables that show high differences in the context-dependent anonymized dataset often exhibit similar deviations in the synthesized dataset. The smaller VKA group shows generally larger differences across both protected datasets. For some attributes, including the Stroke flag in the VKA group, the context-dependent anonymized dataset retains distributions more accurately, while for others, like the Moderate/severe liver disease flag in the VKA group, the synthesized dataset does.

For the plotted attributes, the average relative difference between the protected datasets and the original in the DOAC group was 2.36 for context-independent anonymization, 0.79 for the context-dependent anonymization, and 1.35 for the synthetization. For context-independent anonymization, this was calculated based only on attributes that were not entirely suppressed. In the VKA group, the corresponding differences were 4.88, 1.28 and 2.07, respectively. This indicates that the context-dependent anonymization had the lowest average difference, closely followed by the synthetization. The context-independent anonymization performed the worst, with a considerably higher deviation.

**Fig. 2:**
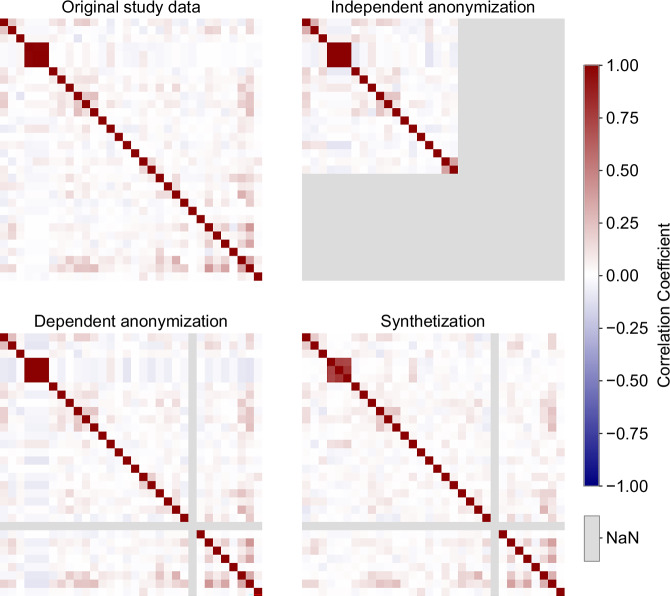
Correlation structure in original and protected datasets. Pearson correlation matrices are shown for the original, anonymized and synthesized datasets to assess preservation of pairwise relationships between attributes.

Investigating the bivariate structural changes of the datasets was done using pairwise Pearson correlations as well as the overall aggregated distance of the correlation coefficients to the original study dataset. The pairwise correlations are shown in Fig. 2 for all 32 numerical attributes (see Supplementary Fig. 1 for correlation-difference matrices). For the context-independent anonymized dataset, correlations could be calculated for only 19 of the 32 attributes, and for these 19 attributes correlations were mostly preserved, as reflected by the average distance of 0.030. The context-dependent anonymization and the synthetization retain most attribute correlations well. This is also reflected in the average correlation distance of 0.019 and 0.027. The figure also shows that context-dependent anonymization and synthetization reduced the number of obesity cases in the original study data (fewer than five) to zero. Therefore, no correlation for this flag could be calculated for both datasets, indicated by the single gray cross in both figures.

To complement the descriptive fidelity analyses, multivariate similarity was evaluated using integrated α-precision (IPα), integrated β-recall (IRβ), and authenticity following previous work^40^. Because these metrics were originally proposed for generative models, they are most naturally interpreted for the synthetic dataset. Table 2 summarizes the results across all datasets. Context-independent anonymization showed the lowest IPα of 0.35 and IRβ of 0.14, indicating limited multivariate overlap with the original data and sparse coverage of its support. Its higher authenticity of 0.72 reflects that heavily transformed records are farther from their original counterparts, but for anonymization, this mainly indicates that records were altered more strongly rather than generative novelty. Context-dependent anonymization retained high IPα of 0.96 and IRβ of 0.86, consistent with only limited transformations being applied to many records. Its low authenticity of 0.14 is expected for an approach that modifies rather than regenerates records. Synthetization achieved high IPα of 0.98, IRβ of 0.44, and authenticity of 0.51, indicating that most synthetic records resembled plausible original records, but covered only part of the variability in the original data. Stratified analyses by treatment group confirmed that these patterns were consistent within both subgroups, as shown in Supplementary Table 3.

**Table 2:** Complementary multivariate similarity of each protected dataset relative to the original, assessed using integrated α-precision (IPα), integrated β-recall (IRβ), and authenticity

Fidelity metric	Independent anonymization	Dependent anonymization	Synthetization
IPα	0.3483	0.9564	0.9826
IRβ	0.1391	0.8571	0.4424
Authenticity	0.7225	0.1440	0.5078

IPα measures the fraction of protected records within the support of the original distribution, with values closer to 1.0 indicating greater overlap. IRβ measures the fraction of original records covered by the protected data’s support, with values closer to 1.0 indicating greater coverage. Authenticity measures the fraction of protected records that are not near copies of original records, with values closer to 1.0 indicating greater separation from original records. All metrics were computed on all numeric columns in the dataset, including binary indicator variables. For context-independent anonymization, suppressed columns were imputed with the original-data column median, creating constant-valued features that may inflate nearest-neighbor distances and can reduce IPα and IRβ.

### Utility evaluation

**Fig. 3:**
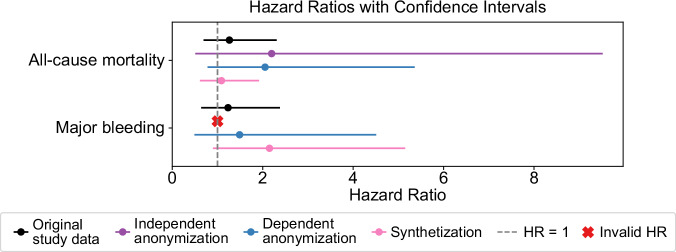
Visualization of hazard ratios and confidence intervals across datasets. Hazard ratios and confidence intervals are visualized for major bleeding and all-cause mortality, displaying the overlap of confidence intervals across original, anonymized and synthetic datasets.

The scientific goal of the original study^2^ was to investigate differences in risks associated with two types of treatment for venous thromboembolisms (VTE). The outcomes investigated were all-cause mortality and major bleeding, which were analyzed using group-weight adjusted hazard ratios (HRs). Covariate balance before and after inverse probability of treatment weighting (IPTW) for each dataset is summarized in Supplementary Table 4. These diagnostics showed that weighting improved within-dataset balance overall in the original and protected datasets. To assess whether anonymization and synthetization affected the study results, these HRs and their confidence intervals (CIs) were compared. Table 3 provides an overview of the number of relevant events within each treatment group, the HRs and CIs for each outcome. Figure 3 visualizes the overlap of confidence intervals across all four datasets.

**Table 3:** Adjusted hazard ratios and confidence intervals across datasets

	Original study data		Independent anonymization		Dependent anonymization		Synthetization	
Outcome	N Events	Adj. HR (CI)	N Events	Adj. HR (CI)	N Events	Adj. HR (CI)	N Events	Adj. HR (CI)
**All-cause mortality**	DOAC = 49	1.26 (.69–2.31)	DOAC = 20	2.20 (.51-9.52)	DOAC = 26	2.05 (.78-5.36)	DOAC = 48	1.09 (.61–1.92)
	VKA = 14		VKA = 2		VKA = 5		VKA = 16	
**Major bleeding**	DOAC = 36	1.23 (.64–2.38)	DOAC = 3	NA	DOAC = 15	1.49 (.49-4.51)	DOAC = 34	2.15 (.9–5.15)
	VKA = 12		VKA = 0		VKA = 4		VKA = 6	

Adjusted hazard ratios (Adj. HR) and confidence intervals (CIs) are reported for major bleeding and all-cause mortality, stratified by DOAC and VKA groups. Hazard ratios were estimated after propensity score based inverse probability of treatment weighting. Values for which hazard ratios could not be calculated are reported as not applicable (NA).

The three protected datasets provided adjusted HRs for the primary clinical outcome of all-cause mortality, ranging from 1.09 for the synthetic data to 2.20 for the context-independent anonymization. The HR of the original study data was 1.26 and therefore in the lower end of that spectrum. Both anonymized datasets showed larger confidence intervals, with the context-independent anonymization showing the largest range from 0.51 to 9.52 compared to the original range of 0.69 to 2.31. These higher CI ranges are directly tied to the smaller number of events in the protected datasets.

Regarding the risk of major bleeding, HRs could not be calculated from the context-independent anonymization due to the absence of events in the VKA group. The HR was slightly elevated with 2.15 in the synthetic data, compared to the 1.23 from the original study data. Here the context-dependent anonymized data provided closer HRs with 1.49.

Overall, all HRs that could be calculated fell within the CI of the original HRs. Anonymization provided slightly larger HRs, due to the smaller number of preserved events. The synthetization provided less consistent results, underestimating the HR for all-cause mortality and overestimating it for major bleeding.

### Privacy risk evaluation

To evaluate the privacy risks, a quantitative assessment was conducted using Anonymeter and shadow model-based membership inference attacks. Anonymeter^25^ was applied in two scenarios: 1) a context-dependent scenario, where only selected attributes were considered to be available to an adversary, and 2) a context-independent scenario, where all attributes were considered for attacks. To provide a baseline comparison, attacks were also conducted on the original study dataset. For shadow-model-based attacks two implementations were used, one specifically developed for synthetic data^24^, and another adapted for anonymized data, referred to as Phantom Anonymization^38^, which closely aligns with the synthetic data shadow model approach.

Table 4 summarizes the average Anonymeter framework risk scores for data linkage attacks and univariate as well as multivariate singling-out attacks. Each attack was repeated 10 times to account for variability, due to the dataset being smaller than recommended. In 9 out of 24 cases (37.5%), all 10 repetitions were classified as invalid by the framework, meaning the main attack did not outperform a naïve baseline attack. These privacy risk scores were still reported but marked accordingly. Importantly, Anonymeter’s privacy risk scores are not raw attack success rates. Instead, they quantify how much better the main attack performs on records used to create the released dataset compared to an analogous control attack evaluated on an independent control set (see Methods section on Privacy evaluation).

**Table 4:** Privacy risk scores for linkage and singling-out estimated with Anonymeter

Privacy attack	Original study data	Independent anonymization	Dependent anonymization	Synthetization
**Context-dependent scenario (lower-risk)**				
Linkage risk	0.81 (0.02)	0.00 (0.00)	0.00 (0.00)*	0.00 (0.00)*
Singling-out univariate risk	0.01 (0.00)*	0.00 (0.00)*	0.00 (0.00)*	0.00 (0.00)*
Singling-out multivariate risk	0.98 (0.00)	0.03 (0.03)	0.03 (0.01)	0.06 (0.03)
**Context-independent scenario (higher-risk)**				
Linkage risk	0.88 (0.01)	0.00 (0.00)	0.00 (0.00)	0.00 (0.00)*
Singling-out univariate risk	0.99 (0.00)	0.00 (0.00)	0.00 (0.00)*	0.21 (0.02)
Singling-out multivariate risk	0.98 (0.00)	0.04 (0.02)	0.06 (0.02)	0.09 (0.03)

Privacy risk scores are reported for both scenarios as mean and standard deviation over 10 iterations per dataset. An asterisk indicates that the framework classified all attacks for that estimate as not valid.

Attacks on original study data in both scenarios resulted in high privacy risk scores, ranging from 0.81 to 0.99, for almost all the attacks. In these cases, the average success rates of the main attacks ranged from 80.6% to 99.5%, while the control attacks ranged from 0.5% to 4.8%, suggesting a large advantage for the main attack over control in these settings. Only the univariate singling-out attack resulted in a privacy risk score of 0.01 and all attacks being marked as invalid, indicating that even against original study data this attack was not successful.

In the context-dependent scenario, both anonymization and synthetization significantly reduced the privacy risks with average risk values ranging from 0.00 to 0.06 for the protected datasets across different attacks. In the context-independent scenario, average risk scores ranged from 0.00 to 0.04 for the context-independent anonymization, 0.00 to 0.06 for the context-dependent anonymization and 0.00 to 0.21 for the synthetization.

Across protected datasets, these low-risk values were generally accompanied by only small absolute improvements in success rates from the control attack to the main attack, whereas the original data showed much larger improvements consistent with substantial leakage. The largest improvement among the protected datasets was observed for univariate singling-out on synthetic data in the context-independent scenario, where the control attack achieved an average success rate of 3.7% and the main attack an average success rate of 23.5%. In most other protected configurations, the improvement over control was close to zero.

**Fig. 4:**
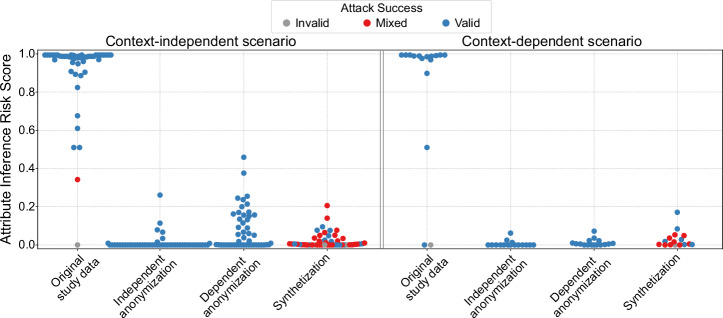
Visualization of attribute inference risks estimated by Anonymeter. Attribute inference risks are shown for the context-independent and context-dependent scenario. Each point in the figure represents the average risk score for a single attribute across 10 repeated attacks. The framework flags unsuccessful attacks, where the risk of a baseline attack exceeds the risk of the actual attack as “invalid”. In the figure attacks are marked as “invalid” if all 10 attacks were invalid (gray) and “valid” if all 10 attacks were valid (blue). Otherwise, the score for the attribute is marked as “mixed” (red).

Figure 4 visualizes the average attribute inference risks for individual attributes as estimated by the Anonymeter framework. Attacks were repeated 10 times and the average results are reported.

For original study data, the average attribute inference risk across attributes was 0.91 in the context-independent scenario and 0.83 in the context-dependent adversary scenario. The protected datasets reduced the average risk across all attributes in the context-dependent scenario to 0.01 for context-independent anonymization, to 0.01 for context-dependent anonymization and 0.03 for synthetization. In the context-independent scenario, the risk was reduced to 0.01 for context-independent anonymization, 0.08 for context-dependent anonymization and 0.02 for synthetization.

When looking at individual attribute risks in the context-independent scenario, especially for context-dependent anonymization, some individual attributes had higher average risk scores, with the highest risk being 0.46 for arterial hypertension. In this case, the main attack achieved an average success rate of 96% compared to 92.6% for the control attack, corresponding to an absolute improvement of about 3.4 percentage points over control. Because Anonymeter normalizes by the remaining headroom to perfect success, the same absolute improvement of the main attack over control can yield a larger risk score when the control success rate is already high. For context-independent anonymization and synthetization, the highest average risk scores for individual attributes were 0.26 and 0.21, respectively, in both cases for the Varicose veins/post-thrombotic syndrome flag. In these cases, the corresponding absolute improvements from control to the main attack remained small: for context-independent anonymization, the main and control success rates were 96.8% and 95.7%, and for synthetization they were 91.6% and 89.4%.

In the context-dependent adversary scenario, attribute inference risks remained low for both anonymizations, with no attribute exceeding a risk score of 0.07. For the synthetization, only the attribute death flag reached 0.17, with all other attributes not exceeding 0.08. In this case, the main and control success rates were 91.7% and 90.3%, corresponding to an absolute improvement of about 1.4 percentage points.

**Fig. 5:**
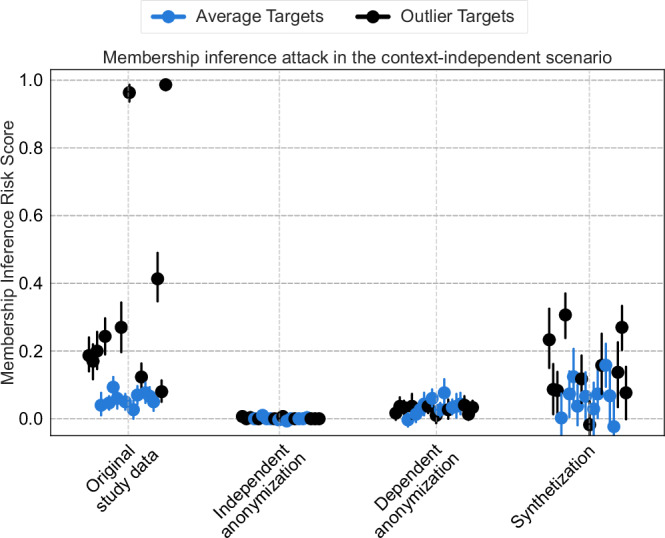
Membership inference risks in the context-independent scenario. Membership inference risk scores are computed in the context-independent scenario using all attributes for the attack and are reported as the attacker’s membership advantage. A privacy risk score of 1 indicates high confidence in predicting that the target was a member of the training set, whereas a privacy risk score of 0 indicates no confidence in predicting whether the target was part of the training set.

Figure 5 presents the results of membership inference attacks for anonymized and synthesized data based on implementations of Stadler et al.^24^ and the Phantom Anonymization Framework^38^. Attacks were evaluated for 10 outlier and 10 average target records, focusing only on the context-independent scenario.

Using the original study data, the results for 20 target records indicate that for some records, particularly outliers, their presence in the training set can be inferred with moderate to high confidence (Privacy Risk Score 0.2–1.0). For average targets, the mean Privacy Risk Score was 0.06, while for outliers it was 0.36. Both context-independent and context-dependent anonymization provided robust protection against our membership inference attack, resulting in mean Privacy Risk Scores of <0.01 and 0.04 for average targets and <0.01 and 0.03 for outlier targets, very close to the optimal outcome of 0. A slightly higher variance was observed in the synthetic data, where outliers showed slightly higher Privacy Risk Scores with a mean score of 0.14 compared to average targets with a mean score of 0.05.

## Discussion

In this study, we evaluated two concrete applications of anonymization and one concrete application of synthetization as protection methods for a small, high-dimensional real-world health claims dataset with the aim of assessing how reproducible the clinical research findings are when using protected datasets. Our comparison of both methods across fidelity, utility and privacy protection showed that each evaluated approach had its distinct advantages. Our results show that both anonymization and synthetization were able to preserve fidelity, while replicating original study results in our utility assessment was only partially successful. Considering the assessment of residual privacy risks, context-independent anonymization offered the strongest privacy protection among the three setups but also performed the worst at fidelity and utility. Context-dependent anonymization and synthetization offered a more balanced compromise between fidelity, utility and privacy protection.

The fidelity assessment showed that all methods were able to preserve distributions and correlations for many attributes that were not entirely removed. The context-independent anonymization suppressed the most attributes and retained individual attribute distributions and correlations worse than the other two setups. Context-dependent anonymization and synthetization were comparable in this regard, both retaining most of the attributes with distributions and correlations comparable to those of the original study data. However, the average scores for context-dependent anonymization were slightly better. By contrast, synthetization was the only method able to provide a dataset with the same number of records as the original dataset, as context-dependent anonymization suppressed 22.8% of records and context-independent anonymization suppressed 36.4% of records. The complementary multivariate similarity metrics showed that context-dependent anonymization and synthetization preserved multivariate distributional properties more consistently than context-independent anonymization. The IPα and IRβ metrics suggested that context-dependent anonymization and synthetization both retained substantial overlap with the original data distribution, whereas context-independent anonymization showed markedly reduced overlap and coverage. Synthetization achieved the highest IPα, indicating that synthetic records largely remained within the support of the original data, but its lower IRβ compared with context-dependent anonymization suggested less complete coverage of the original distribution. However, IPα and IRβ should be interpreted with caution for the anonymized datasets, as these metrics were developed for synthetic data generated by generative models, whereas anonymization by design produces transformed versions of original records. Authenticity also differed strongly between methods and likewise should be interpreted with caution in this comparison, as low authenticity is expected for anonymization approaches that transform original records rather than generate new ones. Stratified analyses by treatment group showed that these multivariate similarity patterns were consistent within both clinically relevant subgroups.

For the utility assessment, even with original study data, the calculation of hazard ratios and confidence intervals was inherently uncertain, due to the small number of events in the data. Examining how anonymization and synthetization influence the resulting estimates remains valuable, as it provides insights into the robustness and interpretability of such methods under realistic data constraints.

The original study did not report a statistically significant difference between the treatment groups. Our results were partially successful in reproducing this outcome with the protected datasets. Except for the major bleeding hazard ratio (HR) for context-independent anonymization, all HRs could be calculated and were within a similar range as the original study, showing no significant statistical difference between the treatment groups. When comparing the confidence intervals, we see larger ranges in the context-independent anonymization, due to the reduction of VKA cases. We believe that the results of the context-independent anonymization in recreating the primary outcomes render it largely impractical for the use case at hand, and the more interesting comparison lies between the context-dependent anonymization and the synthetization. The threat-modeled anonymization provided slightly higher hazard ratios with larger confidence intervals, while the hazard ratios from synthetic data were smaller for all-cause mortality events and larger for major bleeding events than HRs from the original study data. This raises the question of the predictability of derived statistical outcomes from synthetic data and can have implications on how to work with such protected datasets. From a conservative viewpoint, the consistent widening observed with anonymization could be interpreted as an honest reflection of increased uncertainty due to the removal of information, potentially preventing overconfident conclusions. However, it also points to a definitive loss in the ability to make precise inferences. The behavior of synthetic data presents a different challenge, as shifts in HR could lead to different conclusions about the range of plausible effect sizes if not carefully considered.

As both anonymization and synthetization can employ safeguards that affect rare events or categories of data, results that rely on such rare events or categories may not be recreated successfully and are especially vulnerable to utility loss. This was one of the challenges of the selected dataset, as reflected in the skewed number of events for some outcomes and is likely to be improved by a larger dataset size. This suggests that protected datasets that are based on such small high-dimensional datasets might be more suitable for feasibility assessments and method development than for deriving robust clinical insights.

To complement our utility assessment, we empirically evaluated the privacy of each protection method. The results of our baseline privacy assessment on the original study data, which was anonymized for use in a secure processing environment with high organizational safeguards, confirmed inherent privacy risks, demonstrating the need to apply further privacy-enhancing technologies before data publication. All three data protection approaches lowered the investigated privacy risks, with context-independent anonymization leading to the most robust protection across all attacks. While context-dependent anonymization also led to robust protection across most of the attacks, it did exhibit a notable increase in privacy risks for Anonymeter’s attribute inference attack in the context-independent scenario compared to the independent anonymization and synthetization. This again highlights the dependence of context-dependent anonymization on the correctness of the threat modeling. Synthetization also offered robust protection across all but one attack setup. Only in Anonymeter’s multivariate singling-out attack did the synthetic data show a higher-risk score compared to both anonymizations. In the membership inference attack, privacy risk scores for outlier target records from the original dataset were marginally higher when evaluated on the synthetic data than on the anonymized data. This pattern included cases where synthetic data showed elevated risk even when the same records had lower risk in the baseline attack, and we believe this likely reflects statistical variance introduced by the synthetic data generation process rather than a genuine increase in risk. At the same time, in our specific study setting, the practical harm of membership disclosure may be more limited because some attributes available to the attacker may already reveal part of the sensitive context associated with inclusion in the dataset. Nonetheless, we consider membership inference informative as one component of a broader privacy assessment, particularly because in other settings dataset inclusion itself may be sensitive.

Although a relatively weak privacy threshold of 50% maximum re-identification risk was used, our results showed that the protected datasets offered robust privacy protection against the evaluated attacks. The context-dependent anonymization only protected those attributes identified by our risk assessment and hence exhibited more risks in the context-independent scenario. However, identifying direct and indirect identifiers to which protection should be applied is in line with recommendations from regulatory guidelines^14–16^. Nonetheless, relying on such context-dependence has its caveats, as stronger adversaries with access to more or different attributes than expected may be able to overcome the intended protection.

Overall, it is difficult to estimate the privacy-utility trade-off offered by anonymized and synthetic data before applying these methods to generate protected datasets. Different datasets will likely lead to significantly varying results, making utility and privacy assessments necessary. The configuration of the methods will also have an impact on results, and prior work has already shown that a use case-specific configuration for anonymization can significantly improve the accuracy of downstream tasks^41^. Many synthetic data generation approaches, including the approach used in this study, primarily optimize fidelity, while utility is assessed post hoc on downstream tasks. This indirect approach can lead to a suboptimal privacy-utility trade-off, with recent work showing that synthetic data generation can instead optimize task utility directly without maximizing overall fidelity^42^. However, this also means that successful anonymization and synthetization require substantial prior knowledge about the use case and application scenario.

A limitation of our fidelity evaluation is that it is mainly based on selected, interpretable summary statistics (marginals, group-wise prevalence shifts, and pairwise correlations). These make it straightforward to see how anonymization and synthetization affect clinically meaningful quantities (e.g., changes in subgroup distributions and prevalence). However, such summaries provide only a partial view of the protected data and may miss higher-order multivariate dependencies^40^. We additionally evaluated multivariate similarity using integrated α-precision, β-recall, and authenticity as higher-order diagnostics commonly used in synthetic data evaluation. However, these metrics were originally developed for generative models and are therefore more directly interpretable for synthetization than for anonymization, which transforms existing records rather than generating new ones. They should thus be viewed as complementary multivariate diagnostics rather than directly comparable fidelity measures across anonymization and synthetization. For this reason, we additionally evaluated downstream utility in an analysis aligned with the intended use case. Nonetheless, our reported fidelity and utility may not fully represent performance across other metrics and downstream tasks, and the effects of anonymization and synthetic data generation may disproportionately affect rare events or underrepresented subgroups, potentially impacting fairness in downstream analyses^43,44^. Our results should therefore not be interpreted as a comprehensive assessment of utility across all potential claims-based applications. Addressing both richer multivariate fidelity metrics and subgroup-level fairness impacts is an important direction for future work.

For our approach, we relied on empirical metrics for quantifying residual privacy risks, which require several important considerations. First, our privacy assessment highlighted the difficulty of interpreting the results and deriving acceptable risk thresholds from them. While comparative performance is clear, arguing whether protection is sufficient based on individual attack scores is challenging as there are no established standards providing guidance on how to interpret these results, and acceptable risk thresholds will likely be context dependent.

Next, the study relied on a small selection of privacy evaluation frameworks that cover a range of attack types. However, other types of attacks or evaluation frameworks could yield different results since the quantification of privacy risks depends on the conceptual approach and implementation. Several alternative privacy attacks have been proposed^45,46^ and a more thorough comparison of existing tools is needed for future work.

Finally, empirical approaches, such as the ones used here, have been critiqued for potentially underestimating risks, especially for high-dimensional data^47^. We evaluated membership inference only in the context-independent scenario and while this corresponds conceptually to the higher-risk setting, empirical attacks may in practice be more effective when operating on carefully chosen attribute subsets. Developing principled attribute-selection procedures to better navigate such dimensionality effects and strengthen empirical privacy evaluations is an interesting direction for future work. As our membership inference evaluation relied on a limited set of target records, selected to represent average and outlier records according to the distance-based ranking described in previous work^48^, it may underestimate risk if other vulnerable records were not included. Such restriction to a small number of targets is common in computationally expensive privacy evaluations and was consistent with the evaluation frameworks used here. In the unprotected data, this target selection still separated average from outlier records, whereas such differences were no longer apparent after protection. Our findings should therefore be interpreted as empirical results for this specific attack setup, rather than as ruling out stronger membership-inference signals under alternative setups.

In the absence of formal privacy guarantees and standardized benchmarks for synthetic data, empirical privacy risk estimation currently remains the most practical approach for evaluating synthetic data and has been utilized successfully before^25,29,47,49^.

For our anonymization setup, we built on a previous study in which we demonstrated how to systematically determine a suitable anonymization configuration for this dataset based on context-independent fidelity^50^. Synthetic data were generated using the MOSTLY AI framework, selected both for its capabilities (see Methods section on Data synthetization) and because it was already available within our project and allowed direct interaction with its developers. However, further studies are needed to test a more diverse set of anonymization and synthetization strategies. More commonly used synthetization approaches such as CTGAN^28^ or TabDDPM^51^ could yield different results and would be an interesting direction for future work. In particular, synthetization approaches that provide formal privacy guarantees might be of interest. Examples of these approaches include differentially private data synthetization models, such as PATE-GAN and DPGAN^27,52^. These models introduce carefully calibrated noise into model training so that the presence or absence of any single record has only a limited, quantifiable effect on the generated datasets^26^. However, although Differential Privacy is widely regarded as a robust and well-established privacy mechanism, it often entails a considerable loss in data fidelity and utility, particularly for small datasets^53,54^.

In the present study, we primarily focused on highlighting potential issues when using anonymization and synthetization technologies on real-world datasets for medical research and provide practical recommendations. Our results suggest that, at least for small high-dimensional datasets such as ours, clinical evidence should be derived from the original data, even when protected datasets show high structural similarity on standard fidelity measures.

To place these findings in context, we compare them with prior work on anonymized and synthetic clinical data. Beyond our study, a substantial body of work has investigated how anonymized data^6,32,35,41^ and synthetic data^5,17,20–22,37^ can be used in healthcare. These studies typically evaluate either anonymization or synthetization. In contrast, direct comparisons of both approaches on real-world clinical datasets are still relatively rare, but the findings of these studies are broadly consistent with ours. Francis et al.^30^ tried to replicate the key analyses of a cardiorespiratory fitness study using anonymized and synthesized versions of the original dataset (713 records, 8 attributes). They found that outcomes varied by methods, as ARX and SynDiffix preserved the study’s main conclusions, whereas SDV’s CTGAN did not, showing that synthetic generation can distort utility underlining the importance of a utility analysis. To evaluate privacy, they used Anonymeter, and all approaches offered strong protection. Another study by Johann et al.^29^ compared anonymization and synthetization on a real-world cardiology study (2441 records, 18 attributes) and found that both methods resulted in minor utility loss. Privacy was evaluated with Anonymeter and hold-out metrics, showing good protection overall, with anonymization slightly worse on hold-out metrics and synthetization slightly worse on multivariate singling-out risk. Anonymization provided a formal k-anonymity guarantee (*k* = 2), while the synthetic data generation was flexible in producing any number of row counts. They also tested a hybrid approach combining anonymization and synthetization, which offered the best overall balance in their experiments.

While these studies provide valuable insights into the comparison of anonymized and synthesized data, our work extends this comparison to a higher dimensional, health claims dataset (1727 records, 39 attributes). Another distinguishing feature of our approach is the evaluation of two distinct anonymization strategies, context-independent anonymization and context-dependent anonymization. Furthermore, our privacy evaluation included attack scenarios specifically tailored to both the context-independent and context-dependent scenario, yielding additional insights into the robustness of each method against privacy threats under different assumptions of attacker knowledge.

In summary, our work shows that none of the three specific implementations of the protection approaches dominated our three comparison dimensions for this small high-dimensional health claims dataset. The utility assessment could only be partially performed for the context-independent anonymization and even context-dependent anonymization and synthetization showed limitations in the reproducibility and stability of the clinical research results. This reinforces that high structural similarity on summary fidelity measures does not necessarily translate into stable downstream clinical inferences, particularly when outcomes depend on rare events in small, high-dimensional data.

Choosing which method to use will likely depend on the data sharing context and the goals of the data sharing activity and, in some cases, personal or institutional preference. For example, if stronger privacy guarantees are required, a context-independent anonymization approach may be appropriate, for example to provide data for method development. On the other hand, if it is important to retain the original number of records or generating larger datasets, e.g., for testing compatibility with downstream pipelines and the development of methods, synthetization allows for this by generating an arbitrary number of synthetic records.

Our findings underline the importance of investigating the privacy-utility trade-off that protection methods offer, and future research should focus on providing standardized evaluation frameworks and recommendations for protected data. To get a more holistic view of this comparison, further real-world applications of both methods on a more diverse set of datasets are needed. In the meantime, for small high-dimensional datasets such as ours, protected datasets may be most suitable for feasibility assessments and method development, whereas robust clinical evidence should preferably be derived from the original data under appropriate governance and safeguards.

## Methods

### Study data and dataset description

To reproduce the original study by Duros et al.^2^ the InGef research database was used, a healthcare database that at the time of the study contained anonymized claims data from 51 German statutory health insurances, covering roughly 9 million individuals. The database contains demographic, outpatient, inpatient and prescription data, as well as information on sick leave, prescribed aids and remedies and the costs which accrued in the respective sectors^55^. Data are longitudinally linked over a time period of up to ten years. All patient-level and provider-level data in the InGef research database are anonymized and processed in the context of a protected research environment with trusted data users to comply with German data protection regulations and German federal law. Accordingly, this anonymization includes removal of direct identifiers, hash-based pseudonymization of insured-person and provider identifiers, and generalization of selected variables (e.g., reduction of geographic precision, aggregation of practice and hospital identifiers, and transformation of dates to month or quarter).

In the original study, concomitant use of direct oral anticoagulants (DOACs) and antiplatelets was compared with concomitant use of vitamin K antagonists (VKAs) and antiplatelets, using inverse probability of treatment weighting to balance exposure groups^2^. In brief, patients aged 18 years or older with incident venous thromboembolisms (VTE) who initiated treatment with either a DOAC or a VKA while being exposed to antiplatelets were included in the study. The detailed inclusion and exclusion criteria were identical to the original study^2^.

This study investigated two clinical outcomes: major bleeding and all-cause mortality. Major bleeding was defined as an inpatient diagnostic code for bleeding. The covariates used for the propensity score calculation were defined using relevant diagnostic and procedure codes, which were consistent with those used in an original study by the same research group on VTE^56^. The outcomes investigated rely on rare events, which pose a realistic yet challenging scenario for anonymization and synthetization.

The final analysis dataset, which was used for anonymization and synthetization, contained only the actual patient population, structured as one row per individual and consisting of 1727 records with 39 attributes. The dataset included 3 attributes with demographic data (age and gender), 20 with comorbidities, 9 with study information, and 7 with outcome data. An overview of the attributes is provided in the Supplementary Table 1.

### Data sharing context

The InGef dataset consists of anonymized claims data from German statutory health insurances. This prior anonymization was designed with high organizational safeguards in mind, as the data is only accessible to trusted researchers working on approved projects within a secure processing environment. This study explores privacy-enhancing strategies for less controlled data sharing contexts and evaluates privacy under two scenarios that differ only in their assumptions about the auxiliary knowledge of the attacker. In both scenarios, potential re-identification attempts by data recipients are considered.

In the context-dependent scenario, the attacker’s auxiliary knowledge is restricted to the set of attributes identified by the threat modeling.

In the context-independent scenario, a worst-case perspective is adopted in which any attribute in the dataset may be used by the attacker as auxiliary information.

### Threat modeling

The threat modeling, which serves as the initial step of the data anonymization process, was published previously^50^. The modeling consisted of a comprehensive qualitative and semi-quantitative risk assessment to identify attributes with an increased risk of being used in re-identification attacks. The goal of the threat modeling was to categorize all attributes into four mutually exclusive categories: direct identifiers, indirect identifiers, sensitive attributes and insensitive attributes. This categorization guides the configuration of the context-dependent anonymization process, and in this study, it is also used to inform the modeling of the two threat scenarios for the privacy evaluation.

The threat modeling was conducted analogously to the approach of Jakob et al.^57^. Specifically, attributes were marked as direct identifiers if they contained explicitly identifying information (e.g., IDs). All remaining attributes were assessed along the three dimensions proposed by Malin et al.^58^: replicability, availability, and distinguishability. Replicability captures how stable an attribute is for an individual over time; data availability reflects how plausibly the attribute could be known or obtained from external sources; and distinguishability reflects the extent to which an attribute can single out individuals. Each attribute was rated as low/medium/high on each dimension (mapped to 1–3), the ratings were summed per attribute, and the total was compared against a threshold to determine whether the attribute should be treated as an indirect identifier in the anonymization configuration. Remaining, non-identifying attributes were marked as sensitive if their disclosure could reasonably be expected to cause harm or distress to data subjects. We reused the ratings as well as the threshold from the previous publication, where two independent privacy researchers rated each attribute and disagreements were resolved by consensus discussion with a third.

The dataset was categorized into 1 direct identifier, 10 indirect identifiers, 5 sensitive attributes and the remaining attributes were classified as insensitive attributes. The detailed results of the threat modeling are presented in the Supplementary Table 2.

### Data anonymization

The anonymization was performed using ARX^59^ and was based on previous work^50^, which systematically evaluated multiple anonymization strategies and parameterizations for this dataset to balance privacy protection and data fidelity. Two of those setups based on *k*-anonymity^12^ and t-closeness^13^ were adapted, with a refinement to the *t*-closeness configuration. While the original approach utilized hierarchical t-closeness, the anonymization in this study uses equal distance t-closeness, as this led to slightly less utility degradation at a comparative level of privacy. The remaining configuration of our anonymization remains consistent with the previous publication. We used two setups: context-independent anonymization, in which all available variables are protected, and the anonymization is configured to use *k*-anonymity, with *k* = 2 and *t*-closeness with *t* = 0.5; and context-dependent anonymization, in which only the direct, indirect and sensitive attributes identified by the prior risk assessment are protected, and the anonymization is configured to use k-anonymity with *k* = 2 and *t*-closeness with *t* = 0.5.

The anonymization procedure combines generalization, microaggregation, and suppression to satisfy the privacy constraints. Age was generalized into either 5 or 10-year intervals. To preserve data types, generalization intervals were represented as the midpoint value. Birth quarter was represented as the quarter’s starting date and was anonymized via microaggregation by replacing values within each equivalence class with the class-wise mean date. The date of first VTE diagnosis was aggregated analogously. All other date attributes are converted to relative day offsets from the first VTE diagnosis and then anonymized via generalization, using intervals of 30, 90 or 360 days. In postprocessing, all dates were transformed back to concrete dates using the microaggregated first VTE date and the generalized offsets. Where necessary to meet the privacy constraints, ARX was allowed to suppress complete attributes and individual cell values. Given this configuration, ARX searches for a transformation that optimizes a specified fidelity objective under the privacy constraints. In this study, the granularity metric from Appendix D^59^ was used.

### Data synthetization

The synthetic data was created using MOSTLY AI’s open-source Synthetic-Data SDK^39^. The SDK is a toolkit built on a generative, autoregressive deep-learning framework^60^ adapted for tabular data, supporting a variety of feature types. For this study, the SDK’s default settings were used, which produce a statistically representative synthetic version of the input dataset.

The SDK incorporates measures to reduce re-identification and linkage risks^61^. First, data synthesis breaks the one-to-one mapping between original and synthetic records. Second, during model training, the SDK uses early stopping and regularization (including dropout) to mitigate overfitting. This helps ensure the model captures global patterns and correlations rather than record-level details. Third, it protects feature-level outliers by grouping rare categories and clipping extreme numerical values prior to training.

The SDK also provides basic privacy checks that compare the proximity of synthetic–training pairs to synthetic–hold-out pairs^62^. Since the entire dataset was synthesized and protection was applied to all attributes, it could be considered conceptually equivalent to context-independent anonymization.

### Utility and fidelity evaluation

All datasets underwent preprocessing and analysis using a single standardized script to ensure comparability. The pipeline harmonized datasets to a common analysis schema, enforced consistent variable naming, type-casting, and date formats, and derived analytical variables such as follow-up durations and age groupings. In some cases, the original study data contained the same variables in multiple formats (e.g., dates represented both as calendar dates and as days since the first VTE diagnosis). For protected datasets, only one representation was used during anonymization or synthesis, and the other was recalculated during postprocessing.

Finally, R scripts that output fidelity metrics and replicate the results of the original study were used across all datasets. All analyses were conducted using Microsoft R Open software, version 4.0.2. The plots and tables were generated using Python based on the four datasets and the results from these R scripts.

A fidelity assessment was conducted to determine the structural similarity between the protected datasets and the original study dataset, as captured by selected univariate and bivariate summary statistics. This aligns with the concept of context-independence, as we simply analyze general similarity of the protected datasets to the original data, without tailoring this part of the analysis to the actual use case. Descriptive statistics (counts, proportions, means) were calculated for key demographic and clinical variables and compared across datasets. For categorical variables, relative distributions within treatment groups, normalized to each dataset’s total records, were also examined, and deviations were visualized using normalized prevalence plots. For continuous variables, means and standard deviations were compared.

To evaluate bivariate structural similarity, pairwise Pearson correlations between attributes were computed for each dataset, and the average absolute difference from the original dataset’s correlation matrix was calculated. This quantified the extent to which relationships between variables were preserved after anonymization or synthetic data generation.

To complement these descriptive analyses, multivariate similarity was assessed using integrated α-precision, integrated β-recall, and authenticity as proposed in previous work^40^. These metrics were originally developed to evaluate synthetic data generated by generative models and quantify the fraction of protected records within the support of the original data (IPα), the fraction of original records covered by the protected data’s support (IRβ), and the fraction of protected records that are not near copies of original records (authenticity). All metrics were computed on numeric variables after min-max normalization fitted on the original data, with missing values imputed by the original-data column median. The implementation of the metrics follows the implementation accompanying the original publication^40^. Subgroup fidelity was additionally evaluated by repeating the analysis stratified by treatment group, with results reported in Supplementary Table 3.

Data utility was assessed by evaluating the ability to reproduce the analysis results of the original study^2^ using the protected datasets. This aligns with the concept of context-dependence as we analyze the protected datasets using the actual use case. The original study examined differences in all-cause mortality and major bleeding between patients receiving direct oral anticoagulants and vitamin K antagonists for venous thromboembolism.

For each dataset, relevant clinical events were identified and adjusted hazard ratios (HRs) with 95% confidence intervals (CIs) were calculated using weighted Cox proportional hazards models. Propensity score–based inverse probability of treatment weighting was applied to account for baseline differences between treatment groups. Covariate balance before and after weighting was assessed using standardized mean differences for which detailed balance diagnostics are provided in Supplementary Table 4. HRs and CIs from each protected dataset were compared with those from the original study dataset to evaluate the extent to which anonymization and synthetic data generation altered effect estimates.

### Privacy evaluation

The level of privacy protection provided by the different data protection methods was empirically evaluated. The risk associated with a single protected dataset release was considered. Knowledge of the protection mechanism and access to reference data from the same population were assumed for the adversary. Three privacy assessment approaches were applied: Anonymeter framework attacks^25^, shadow model–based membership inference attacks for synthetic data as described by Stadler et al.^24^, and Phantom Anonymization^38^, an anonymization-specific adaptation of the shadow model method. For all assessments, black-box access to the anonymization or synthetization process was sufficient, meaning the procedure could be executed on reference data using identical parametrization, while access to internal parameters such as trained model weights was not assumed.

The shadow model-based approaches required that an adversary possess a dataset drawn from the same population as the original data, whereas the Anonymeter framework required a split of the original data into training and test sets. Accordingly, only a subset of the original dataset was used for protected data generation, with the remaining data reserved for privacy attacks.

For the experiments with the Anonymeter framework, two context scenarios were considered, the context-dependent scenario, where only a subset of attributes was used for attacks, and the context-independent scenario, where all attributes were used for attacks. In both scenarios, linkage, singling-out (univariate and multivariate), and attribute inference risks were evaluated using Anonymeter’s attack-based evaluations.

In brief, for each risk Anonymeter runs three variants: a main attack evaluated on training records used to generate the protected dataset, a naïve baseline attack that performs a random-guessing based attack, and a control attack that repeats the main attack but evaluates success on an independent control dataset. The reported risk score captures training-specific leakage by normalizing the excess success of the main attack over the control attack. Formally, risk is computed as $R=\frac{{r}_{{train}}-{r}_{{control}}}{1-{r}_{{control}}}$, where ${r}_{{train}}$ is the success rate of the main attack, and ${r}_{{control}}$ is the success rate of the control attack.

Singling-out predicates are derived from the released dataset (univariate: unique values and min/max-based thresholds; multivariate: conjunctions across multiple attributes). A singling-out guess is counted as correct if the predicate matches exactly one record in the attacked original dataset (training or control). Linkage is assessed via k-nearest-neighbor matching across two disjoint attribute sets, counting a link as successful if the resulting neighbor sets overlap. Attribute inference predicts a secret attribute from nearest neighbors in the released dataset, using exact match for categorical secrets and a tolerance-based matching for numerical secrets.

In Anonymeter, each attack is evaluated using a random sample of targets per run. In our setup, approximately 20% of the available records (346 of 1727) were used for all attacks, except for the computationally heavier multivariate singling-out attack, for which the number of queries was capped at 100. Evaluations were repeated 10 times per dataset to account for stochasticity in Anonymeter’s attack sampling, and mean privacy risk scores with standard deviations were reported. Attacks were classified as invalid when they did not outperform the naive baseline attack; these cases were still reported but flagged accordingly.

Shadow model–based membership inference was only evaluated in the context-independent scenario and specific target records from the dataset were selected, as performing the attack on all records was computationally infeasible. We restricted membership inference to the context-independent setting, as it represents the strongest attacker knowledge assumption.

In brief, the membership inference for synthetization works by training multiple instances of the synthetic data generator on reference datasets from the same population, alternately including and excluding the target record. The attacker generates protected datasets from these generators to obtain labeled examples (target included vs excluded). The attacker then trains a classifier to distinguish these datasets, and applies this classifier to infer whether a target record was present in the training data used to generate the released dataset. The Phantom Anonymization framework works analogously, with the difference that the anonymization of the reference datasets can be performed directly (i.e., without training a generative model as needed for synthetization).

Both approaches were configured to use the random forest classifier together with the attribute-wise marginal distribution feature set (binned counts for continuous attributes and category frequencies for categorical ones) to predict whether the target record was included in the training set used to generate the protected dataset. Target selection followed a previously proposed method for identifying “average” and “outlier” records^48^. A total of 10 average and 10 outlier targets were evaluated for each dataset. Following the coverage-based rationale in Meurers et al.^38^, sample sizes and repetition counts were chosen such that each record in the attacker reference pool had at least 95% probability of being included at least once across the repeated draws. In this setting, the criterion was satisfied by using samples of 500 records with 10 training and 10 test replicates per run. Thirty independent runs were performed, exceeding the minimum implied by the high-overlap scenario, to reduce variance.

The reported membership inference risk score was computed based on the attacker’s true-positive rate (TPR) and false-positive rate (FPR) for inferring whether a target record was included in the training set. The score was calculated as ${\rm{R}}={\rm{TPR}}-{\rm{FPR}}$. A value of ${\rm{R}}=0$ indicates no advantage over random guessing while values close to 1 indicate near-perfect inference. This score was defined as the complement of the privacy gain reported in the original works for both frameworks^24,38^ and was complemented to align directionality with the other privacy evaluations, for which higher values indicated higher risk. Negative estimates could be observed when the false-positive rate exceeded the true positive rate due to evaluation randomness or a non-optimal learned classifier. In practice, such cases were interpreted as indicating no effective attacker advantage.

To allow comparison of the privacy protection provided by anonymized and synthesized datasets, all attacks were applied to both protection methods as well as to the original study dataset to provide a baseline.

## Supplementary information

Supplementary information

## Data Availability

The data used in this study cannot be made publicly available in the manuscript, the additional files, or in a public repository due to German data protection laws (Bundesdatenschutzgesetz) and legal reasons. In Germany, the utilization of health insurance data for scientific research is regulated by the Code of Social Law. Researchers must obtain approval from the health insurance providers as well as their responsible authorities. As this approval is given only for a specific research question for a specific time and for a specific group of researchers, data cannot be made publicly available. To facilitate the replication of results, anonymized data used for this study are stored on a secure drive at the Institute for Applied Health Research Berlin GmbH (InGef). Access to the raw data used in this study can only be provided to external parties under the conditions of a cooperation contract and can be accessed upon request, after written approval (info@ingef.de), if required.

## Code availability

We release the components of our workflow that can be shared without risk of disclosing sensitive implementation details at: https://github.com/BIH-MI/safe-data-for-safe-medication. This repository includes reference implementations to reproduce the evaluation metrics, the configurations used to anonymize the data and configurations for privacy risk assessments. External frameworks used for anonymization, synthetization and privacy assessments are cited in the manuscript and referenced in the repository. Version information is provided in the repository.
