# Supplementary material for: A case study comparing anonymized and synthetic health insurance claims data for medication safety assessments
Source: NPJ Digit Med. 2026 Apr 13;9:321. doi: 10.1038/s41746-026-02622-5 (PMC13087206; doi:10.1038/s41746-026-02622-5)
Supplement: Supplementary file 1 — Supplementary information [file 41746_2026_2622_MOESM1_ESM.pdf]

Supplementary Figure 1: Correlation-difference matrices relative to the original dataset. Heatmaps show the difference in pairwise Pearson correlations between each protected dataset and the original study dataset (protected minus original). Values close to 0 indicate that correlations are preserved, while larger absolute values indicate stronger deviations from the original correlation structure. The figure is intended as a global structural comparison; individual attribute labels are omitted for readability.

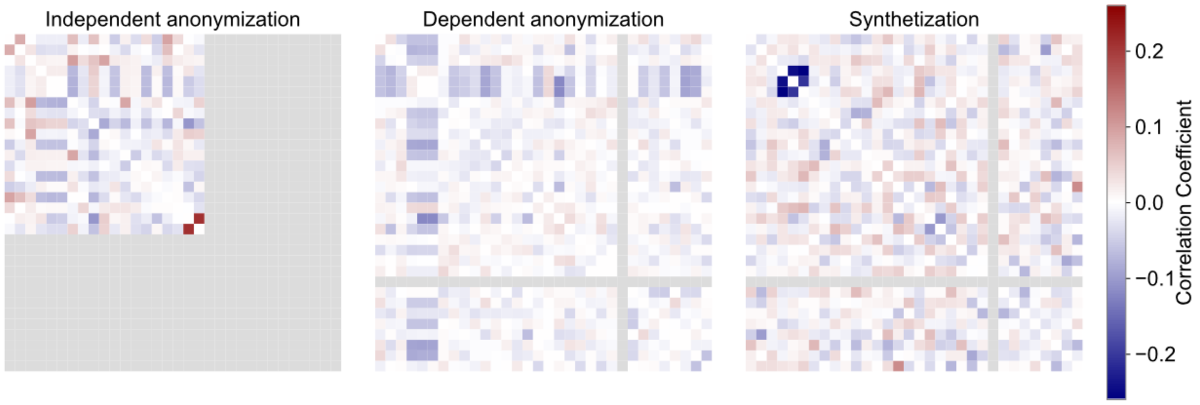

Supplementary Table 1: Attributes of our use case dataset to be considered for anonymization and synthetization. In total the dataset consists of 4 numeric attributes, 2 categorical attributes, 8 dates and 25 binary attributes. For non-binary attributes the data type is reported in brackets after each mention.

Category	*Characteristics*
Demographic data (3)	Age (numeric), Birth quarter (date), Female sex
Comorbidities (20)	Arterial hypertension, Bleeding, Cancer (excl. non-melanoma skin cancer), Chronic kidney disease, Congestive heart failure, Diabetes mellitus, Fracture, Inflammatory bowel disease, Major surgery, Myocardial infarction, Moderate/severe liver disease, Non-steroidal anti-inflammatory drug, Obesity during baseline, Oral contraceptives, Proton pump inhibitors, Selective serotonin reuptake inhibitors, Stroke, Systemic corticosteroids, Tamoxifen, Varicose veins/post-thrombotic syndrome
Study information (9)	Baseline end date (date), Baseline start date (date), Number of days of continuous antiplatelet use before cohort entry (numeric), Overall number of hospitalizations (numeric), Overall number of non-antithrombotic medication (All drugs except B01A) (numeric), Specific anticoagulant drug that patient received (categorical), Anticoagulant treatment group, Patient ID (categorical), Date of first VTE diagnosis (date)

Outcome data (7)	Follow up end date for bleeding outcome (date), Follow up end date for death outcome (date), Date of death (date), Bleeding flag, Death flag, Follow-up end date (date), Recurrent VTE flag

To avoid disclosing dataset-specific extremes, we report only coarse value ranges and the temporal granularity provided in the source data. Age is measured in years (approximately 30–90+). Birth quarter corresponds to the birth date generalized to the start of the quarter. All event dates are generalized to the month and mostly include dates within the 2012–2020 timespan. Count variables (e.g., Overall number of hospitalizations) are non-negative integers smaller than 100. Days of continuous antiplatelet use before cohort entry is an integer and ranges up to roughly 360 days. The specific anticoagulant drug is a categorical attribute with 6 categories.

Supplementary Table 2: Categorization of attributes after threat modelling.

Category	*Characteristics*
Direct identifier (n=1)	Patient ID
Indirect identifier (n=10)	Age, Birth quarter, Female sex, Obesity during baseline, Follow up end date for bleeding outcome, Follow up end date for death outcome, Date of death, Death flag, Follow-up end date, Date of first VTE diagnosis
Sensitive attributes (n=5)	Congestive heart failure, Myocardial infarction, Moderate/severe liver disease, Selective serotonin reuptake inhibitors, Stroke
Insensitive attributes (n=23)	Arterial hypertension, Bleeding, Cancer (excl. non-melanoma skin cancer), Chronic kidney disease, Diabetes mellitus, Fracture, Inflammatory bowel disease, Major surgery, Non-steroidal anti-inflammatory drug, Oral contraceptives, Proton pump inhibitors, Systemic corticosteroids, Tamoxifen, Varicose veins/post-thrombotic syndrome, Baseline end date, Baseline start date, Number of days of continuous antiplatelet use before cohort entry, Overall number of hospitalizations, Overall number of non-antithrombotic medication (All drugs except B01A), Specific anticoagulant drug that patient received, Anticoagulant treatment group, Bleeding flag, Recurrent VTE flag

Supplementary Table 3: Integrated α-precision, integrated β-recall, and authenticity stratified by treatment group (DOAC vs. VKA). The results show that the multivariate similarity patterns observed globally were broadly consistent within both treatment groups.

**Fidelity metric**	**Independent anonymization**	**Dependent anonymization**	**Synthetization**
IPα (VKA)	0.3379	0.9462	0.9631
IPα (DOAC)	0.3482	0.9685	0.9828
IRβ (VKA)	0.1737	0.8660	0.4700
IRβ (DOAC)	0.1350	0.8672	0.4250
Authenticity (VKA)	0.6330	0.1671	0.5310
Authenticity (DOAC)	0.7526	0.1333	0.5146

Supplementary Table 4: Summary of covariate balance before and after IPTW. Absolute standardized mean differences (|SMD|) were used to assess covariate balance between treatment groups before and after weighting. For each dataset, the table reports the number of covariate rows with |SMD| > 0.1 before and after IPTW, as well as the median and maximum post-weighting imbalance. Unique covariates refer to distinct baseline variables included in the balance assessment, namely all comorbidities, “Female sex”, “Age in years”; “Number of days of continuous antiplatelet use before cohort entry”, “Overall number of hospitalizations” and “Overall number of non-antithrombotic medication (All drugs except BA)”.

**Dataset**	**Unique covariates**	**Rows with |SMD| > 0.1 before weighting**	**Median |SMD| before weighting**	**Rows with |SMD| > 0.1 after weighting**	**Maximum |SMD| after weighting**	**Median |SMD| after weighting**
Original study data	25	8	0.0603	1	0.1224	0.0089
Independent anonymization	13	9	0.1272	0	0.0996	0.0467
Dependent anonymization	23	4	0.0597	1	0.1511	0.0101
Synthetization	25	11	0.0690	1	0.1893	0.0130

IPTW improved covariate balance markedly in all datasets. After weighting, residual imbalance was minimal overall, with only 0-1 covariate rows per dataset remaining above the |SMD| threshold of 0.1. Median post-weighting |SMD| values were low across all datasets, further indicating good overall balance after weighting. Because privacy protection reduced the number of available covariates in anonymized datasets, these results should be interpreted primarily within datasets rather than as between-dataset rankings.
